# FT-IR characteristics, phenolic profiles and inhibitory potential against digestive enzymes of 25 herbal infusions

**DOI:** 10.1038/s41598-022-10669-z

**Published:** 2022-04-22

**Authors:** Prinya Wongsa, Posathon Phatikulrungsun, Sasithon Prathumthong

**Affiliations:** Food Science and Technology Program, School of Agro-Industry, Mae Fah Luang University, Chiang Rai, 57100 Thailand

**Keywords:** Biochemistry, Chemical biology

## Abstract

The present study aimed to analyse the FT-IR vibrational characteristics and concentration of phenolic compounds in 25 herbal plants using Fourier transform infrared (FT-IR) spectroscopy and Ultra-High Performance Liquid Chromatography (UHPLC) techniques, respectively, and to evaluate its in vitro potential to inhibit enzymes related type 2 diabetes and obesity. The vibrational spectra regions—3400–3200 cm^−1^—indicated hydroxyl group (O–H) and H-bonded stretching, which is characteristic of polyphenolic compounds. A wide range in phenolic compounds was found among the samples. Caffeic acid is the predominant phenolic compounds in the samples. Total phenolic content ranged from 5.02 mg GAE/g DW to 102.39 mg GAE/g DW. A moderate correlation (*R*^2^) between antioxidant activity and α-amylase inhibition was 0.46, (*p* < 0.05) while that (*R*^2^) of *p*-coumaric acid and α-glucosidase inhibition was 0.54, (*p* < 0.05). Moreover, the herbal infusions showed potential to inhibit digestive enzymes, the highest being on the infusion based on a cup-serving basis.

## Introduction

Reactive oxygen species (ROS) including hydrogen peroxide (H_2_O_2_), superoxide ion (O_2_^−^), and hydroxide radical (OH^−^) may be procured in the human body during normal cellular activities and may cause a broad range of damage to biological systems. In addition, oxidative stress plays an important role in many chronic and degenerative diseases, such as diabetes mellitus, cardiovascular diseases, cancer, and ageing^1^.

Herb and medicinal plants have been used to prepare infusions, so-called “polyphenolic beverages”^2^. Traditionally, herb and medicinal plants are sun or hot-air dried prior to being packed into a tea bag. Major phenolic acids identified in herbal plant species including *Labiatae, Compositae, Umbelliferae, Asteracae, Polygonacae and Myrtacae* were caffeic, *p*-coumaric, ferulic and neochlorogenic, while predominant flavonoids were quercetin, luteolin, apigenin, kaempferol and isorhamnetin^3^.

Fourier transform infrared (FT-IR) spectroscopy is one of the most important non-destructive analytical techniques used to identify the functional groups of chemical constituents and widely used for quality control in the food and beverage and pharmaceutical industries^4^. Recently, FT-IR spectroscopy has developed quickly due to its low noise, rapid speed, high repeatability, easy operation, low expense, and so on. FT-IR has become increasingly useful in the field of evaluating herbal qualities^5^.

Alpha-amylase and alpha-glycosidase enzymes are important enzymes involved in maintaining blood sugar levels, which is one of the therapeutic approaches for type II diabetes and can be achieved by retarding carbohydrates digesting enzymes and glucose absorption through the inhibition of pancreatic α-amylase and intestinal α-glucosidase in the digestive tract, which results in the prevention of excess glucose absorption^6,7^.

In addition, obesity is an excessive accumulated fat in our body and is now considered a global problem by the WHO. It may have deleterious health effects including chronic diseases and disabilities such as hypertension, type II diabetes, coronary artery disease, and stroke^8^. Lipase enzyme digests dietary fats into fatty acids and glycerol^9^. The inhibition of lipase activity is an alternative approach of treating obesity.

Medicinal plants have been defined as plants that contain properties or compounds that can be used for therapeutic purposes or those that synthesize metabolites to produce useful drugs^10^. Several scientific reports have suggested that plant-derived compounds provide biological effects such as prevention of oxidative stress -related diseases due to these activities have been traced to plants’ polyphenol compounds^6,7^.

The objectives of this study were undertaken to determine the qualitative and quantitative characteristics and content of bioactive compounds in indigenous herbal teas commonly consumed by FT-IR spectroscopy and UPLC chromatographic methods, respectively and to evaluate the in vitro enzymatic inhibition against key digestive enzymes relevant to type II diabetes and obesity management.

## Results and discussion

### FT-IR overall characteristics

The vibrational spectrum of a chemical molecule is considered to be a unique physical property. Absorption spectra of dried herbal extracts obtained in the range 4000–400 cm^−1^ are shown in Table 1. The spectra show characteristic peaks. The absorption spectra of dried herbal extracts were similar in the range of 4000 to 1800 cm^−1^. The most intense band of 25 samples was observed at a range of between 3375 and 3320 cm^−1^. The region of 3400–3200 cm^−1^ indicates a symmetric (sym) and asymmetric (asym) stretching of polymeric hydroxyl group (O–H), H-bonded stretching, which is characteristic of polyphenolic compounds (Table S2)^14,15^. In the region of 2940–2925 cm^−1^, the –CH, –CH_2_ and –CH_3_ stretching vibrations, derived from carbohydrates and sugars in dried herbal tea extracts, can be seen^16^. The C–H and C=C–C ring-related vibrations showed the existence of one or more aromatic rings in a structure of chemical compound. The stretching of the C–H and C=C–C aromatic bond appears in the region of 1615–1580 cm^−1^. In addition, the phenolic C–O stretching was observed at ~ 1200 cm^−1^. This stretching is due to the C–O of pyran, typical of flavonoid C-rings^16^. These group frequencies are highly related to the presence of aromatic compounds. The region ranged from 1400 to 900 cm^−1^, is commonly called the fingerprint region because of the large amount of characteristic single bands of low intensities attributed to specific functional groups. Among these groups, C–H, C–O, C–N and P–O bonds are included^14^. However, the chemical compositions of the 25 herbal samples were very complex and diverse and the chemical composition fingerprints strongly overlapped, so identifying the differences between the components of these samples was challenging.

**Table 1:** FT-IR spectra analysis of dried herbal plant extracts.

Herbs	Sample code	Absorption spectrum, wavenumber (cm^−1^)							
		3375–3260	2940–2925	1785–1640	1640–1600	1600–1300	1450–1050	1080–620	935–710
Pandan	H1	3301.78	2931.09	1756.52	1599.01	1452.01	1237.54	774.67	
						1333.02	1114.71		
Stevia	H2	3351.2	2926.54	1717.87	1606.33	1518.87	1306.28	1079.71	
						1407.78	1281.64	925.60	814.96
								897.27	779.64
Lemongrass	H3	3319.53	2939.88				1145.48	926.48	828.39
Asiatic pennywort	H4	3314.97	2940.28		1624.03	1429.08	1241.51	1082.88	773.67
Jiaogulan	H5	3280.13	2929.93			1587.31	1434.92	1079.36	
							1269.84		
Kariyat	H6	3312.38	2932.07	1734.36	1612.44	1400.28	1110.87	826.11	766.98
Mulberry leave	H7	3291.10				1585.83	1435.96	996.4	745.55
							1157.43	935.91	
Cat’s whiskers	H8	3346.06	2938.26	1603.68		1526.73	1272.58	1073	871.88
						1411.74	1146.35	925.08	780.43
Bamboo grass	H9	3291.99	2933.11			1595.13	1438.92	1082.15	
Sea holly	H10	3318.02	2936.3			1598.23	1425.13	1079.36	773.67
Stonebreaker	H11	3304.03		1713.45	1602.8	1396.16	1227.28		Stonebreaker
Jewel vine	H12	3318.74	2936.3		1609.53	1431.74	1146.01		
Safflower	H13	3314.74	2932.54		1600.03	1515.55	1075.79	768.45	
						1423.98			
Chrysanthemum	H14	3374.35	2924.58	1733.45		1438.13	1247.09		
Roselle	H15	3280.23		1784.12		1415.87	1219.94	1101.58	
Butterfly pea	H16	3320.10	2936.36		1606.34	1409.53	1060.34		
Beal fruit	H17	3328.08	2931.62		1604.13	1518.94	1434.55	1085.86	
							1269.15	893.32	
Indian gooseberry	H18	3294.73		1726.16	1609.27	1333.02	1114.71	774.67	
							1237.54		
Bitter gourd	H19	3261.34				1587.3	1400.9	1107	
Siamese senna	H20	3313.62			1602.87	1524.28	1270.31		
						1412.47			
Chinese liquorice	H21	3330.44	2936.5		1604.31	1515.86	1073.02	945.76	935.91
						1419.76			
Alexandria senna	H22	3317.81	2941.55		1596.14	1431.37	1120.03		744.11
Ginger	H23	3291.36	2931.56		1638.76	1403.96	1051.53	929.12	782.17
Ginkgo	H24	3307.89	2936.41	1702.13		1598.92	1250.42		768.3
						1435.24	1111.98		
Black galingale	H25	3334.17	2932.54		1636.87	1515.85	1420.00	1079.81	935.91

**Figure 1:**
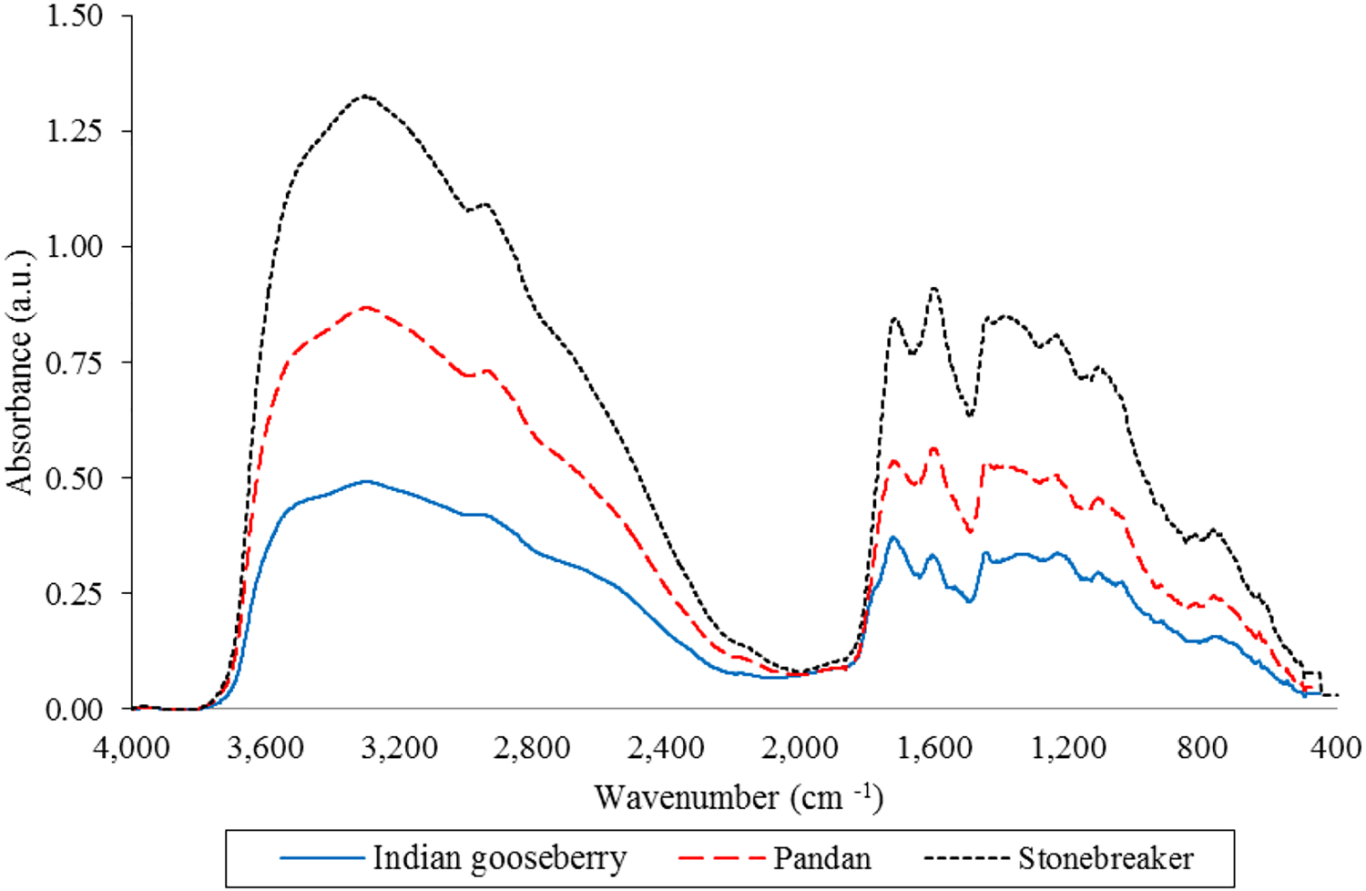
FT-IR spectra for similar three main dominants in pandan, stonebreaker and Indian gooseberry.

Furthermore, the dried pandan, Indian gooseberry and stonebreaker extracts showed similar three main dominant-vibrational spectra of chemical compositions. The peaks at 3296, 2933, 1719, 1594, 1448, 1357, 1100 and 744 cm^−1^ were identical in dried pandan, Indian gooseberry and stonebreaker extracts (Fig. 1). These peaks attributed to phenolic and organic compounds^4^. The most prominent signals for the three dried herbal extracts were the major absorption band (Fig. 1) around 3300 cm^−1^, which can be associated with O–H stretching and C–H stretching vibrations The peaks between 700 and 1800 cm^−1^, the fingerprint zone, could be attributed to C=C–C aromatic ring stretching (1580–1615 cm^−1^, 1450–1510 cm^−1^) and several aromatic out-of-plane C–H (670–900 cm^−1^) and in-plane (950–1225 cm^−1^) bending^14–16^. In addition, peaks for water were observed in the range 1640 cm^−1^ and 3300 cm^−1^ on the basis of functional group H and OH. Protein was observed in the range from 1600 to 1700 cm^−1^ and 1550 to 1570 cm^−1^ on the basis of bond amide I and amide II, respectively. Fat was also observed within these ranges but on the basis of C–H bond and starch was observed in the range from 2800 and 3000 cm^−1^ (C–H stretch region) and in the range 3000 and 3600 cm^−1^ (O–H stretch region).

### Phenolic profile

Plants contain various secondary metabolites, including phenolic compounds, nitrogen compounds, terpenoids and some other endogenous metabolites. The content of phenolic compounds is shown in Table 2. The retention times for gallic acid, (+)-catechin, caffeic acid, *p*-coumaric acid, ferulic acid and quercetin were 1.778, 3.189, 3.393, 3.780, 3.885 and 4.735 min, respectively. The content of caffeic acid ranged from 0.60 mg/g DW (mulberry leave) to 38.57 mg/g DW (Alexandria senna) with the average 3.98 mg/g DW among 25 studied herbal plants. Moreover, the highest content of *p*-coumaric was observed in Chinese licorice (9.36 mg/g DW), while the content of *p-*coumaric was not detected in roselle. It was found that Siamese senna (28.32 mg/g DW) has the highest content of ferulic, followed by Alexandria senna (21.37 mg/g DW and pandan (8.49 mg/g DW). The concentration of 0.28 mg/g DW ferulic acid was observed in pandan extract^17^. Ferulic acid was not detected in roselle.

**Table 2:** The contents of phenolic compounds of dried herbal plant extracts.

Herbs	Phenolic compounds (mg/g DW)					
	Caffeic	*p*-Coumaric	Ferulic	Gallic	Catechin	Quercetin
Pandan	3.91 ± 0.23 e	3.05 ± 0.88 e	8.49 ± 2.07 c	3.35 ± 0.01 c	0.05 ± 0.01 ij	0.04 ± 0.00 c
Stevia	1.83 ± 0.06 gh	0.81 ± 0.02 ghij	1.22 ± 0.04 fgh	n.d	0.01 ± 0.00 k	0.07 ± 0.01 b
Lemongrass	0.70 ± 0.04 lm	2.19 ± 0.06 f	0.85 ± 0.02 fgh	0.83 ± 0.05 h	0.05 ± 0.04 ij	0.02 ± 0.00 cd
Asiatic pennywort	0.94 ± 0.01 jklm	0.62 ± 0.00 ij	1.27 ± 0.05 fgh	n.d	0.03 ± 0.00 jk	0.01 ± 0.00 d
Jiaogulan	1.97 ± 0.31 g	0.79 ± 0.02 ghij	1.79 ± 0.08 fgh	1.19 ± 0.02 fg	0.02 ± 0.00 k	n.d
Kariyat	1.86 ± 0.97 gh	0.92 ± 0.01 ghij	1.05 ± 0.02 fgh	n.d	0.19 ± 0.00 e	0.08 ± 0.00 b
Mulberry leave	0.60 ± 0.02 m	0.45 ± 0.04 jk	0.50 ± 0.06 gh	n.d	0.16 ± 0.02 f	0.01 ± 0.00 d
Cat’s whiskers	0.72 ± 0.01 lm	0.83 ± 0.03 ghij	2.17 ± 0.02 fg	n.d	0.01 ± 0.00 k	0.01 ± 0.01 d
Bamboo grass	1.25 ± 0.03 ijkl	0.70 ± 0.01 hij	1.65 ± 0.01 fgh	n.d	0.07 ± 0.00 hi	0.01 ± 0.07 d
Sea holly	1.33 ± 0.01 hijk	0.78 ± 0.02 ghij	0.86 ± 0.02 fgh	n.d	0.02 ± 0.01 k	n.d
Stonebreaker	4.49 ± 0.49 d	7.55 ± 1.35 b	1.93 ± 0.06 fg	15.00 ± 0.08 a	3.65 ± 0.02 a	n.d
Jewel vine	0.79 ± 0.01 klm	3.07 ± 0.08 e	1.34 ± 0.01 fgh	1.11 ± 0.07 g	0.35 ± 0.00 d	n.d
Safflower	7.40 ± 0.21 c	0.51 ± 0.02 jk	0.81 ± 0.10 fgh	0.87 ± 0.01 h	0.02 ± 0.00 k	0.04 ± 0.04 c
Chrysanthemum	1.40 ± 0.60 ghij	1.42 ± 0.02 g	5.37 ± 0.43 d	0.88 ± 0.00 h	0.09 ± 0.00 gh	0.03 ± 0.00 cd
Roselle	1.72 ± 0.02 ghi	n.d	n.d	1.54 ± 0.01 e	0.11 ± 0.00 g	n.d
Butterfly pea	4.94 ± 0.06 d	4.99 ± 0.22 c	3.74 ± 0.07 e	2.67 ± 0.42 d	0.02 ± 0.00 k	n.d
Beal fruit	1.97 ± 0.31 g	0.79 ± 0.02 ghij	1.79 ± 0.08 fgh	1.19 ± 0.02 fg	0.02 ± 0.00 k	n.d
Indian gooseberry	3.32 ± 0.07 f	5.20 ± 0.04 c	2.49 ± 0.02 ef	5.05 ± 0.03 b	0.05 ± 0.02 ij	0.01 ± 0.00 d
Bitter gourd	1.23 ± 0.20 ijkl	1.22 ± 0.01 ghi	0.92 ± 0.03 fgh	1.18 ± 0.01 fg	0.03 ± 0.00 jk	n.d
Siamese senna	14.06 ± 0.26 b	1.30 ± 0.01 gh	28.32 ± 3.99 a	0.88 ± 0.08 h	0.15 ± 0.01 f	0.01 ± 0.00 d
Chinese liquorice	1.33 ± 0.02 hijk	9.36 ± 0.37 a	2.10 ± 0.05 fg	1.30 ± 0.16 f	0.06 ± 0.01 i	0.01 ± 0.00 d
Alexandria senna	38.57 ± 1.17 a	3.76 ± 0.35 d	21.37 ± 0.01 b	1.30 ± 0.07 f	0.02 ± 0.00 k	0.02 ± 0.00 d
Ginger	1.22 ± 0.00 ijkl	0.89 ± 0.00 ghij	0.92 ± 0.01 fgh	1.20 ± 0.10 fg	2.20 ± 0.00 b	2.30 ± 0.02 a
Ginkgo	0.86 ± 0.01 jklm	2.07 ± 0.00 f	1.73 ± 0.05 fgh	0.89 ± 0.01 h	0.02 ± 0.00 k	n.d
Black galingale	1.85 ± 0.03 gh	0.66 ± 0.01 hij	1.52 ± 0.04 fgh	n.d	0.02 ± 0.00 k	n.d

Values are means ± SD of three measurements (n = 3).

n.d. = not detected.

Means in columns with superscript letters indicate significant differences (*p* < 0.05).

Furthermore, quercetin and catechin are flavonoids. The (+)-catechin content ranged from 3.65 mg/g DW (stonebreaker) and 0.01 mg/g DW (stevia and Cat’s whiskers). The highest quercetin content was observed in ginger (2.30 mg/g DW) while it was not found in beal fruit, jiaogulan, roselle, butterfly pea, ginkgo, sea holly, bitter gourd, stonebreaker, black galingale and jewel vine infusions. The contents of phenolic compounds indicated that the differences in chemical compositions of herbal infusions are due to variations in the plants.

### Total phenolic content

The total phenolic content in the studied herbal infusions, which are prepared per cup serving, is shown in Table 3. A significant difference (*p* < 0.05) in total phenolic content was observed. The contents of polyphenol ranged from 102.39 mg GAE/g DW (stonebreaker) to 5.02 mg GAE/g DW (jiagulan). The difference and average value were 20-fold and 31.02 mg GAE/g DW, respectively. The significantly high content of polyphenol was observed in beal fruit (101.38 mg GAE/g DW), Indian gooseberry (81.86 mg GAE/g DW) and Siamese senna (64.74 mg GAE/g DW). Verma, Rai, and Kaur reported a total phenolic content 72.45 mg GAE/g DW in Indian gooseberry extract^18^ while those of infusions obtained from the *Lamiaceae* family members, including lemon balm, common basil, ranged from 29.39 to 65.38 mg GAE/g DW^19^. In addition, the total phenolic content in the popular herbal infusions including calendula (*Calendula officinalis* L.), lady’s-mantle (*Alchemilla vulgaris* L.), yarrow (*Achillea millefolium* L.), peppermint (*Mentha* × *piperita* L.) and bellis (*Bellis perennis* L.), widely used for herbal teas, ranged between 1.36 mg GAE/g DW and 8.02 mg GAE/g DW^20^.

**Table 3:** Total phenolic content, total flavonoid content and DPPH scavenging activity in herbal tea extracts.

Herbs	Total phenolic content (mg GAE/g dw)	Total flavonoid content (mg QE/g dw)	DPPH radical scavenging activity (%)
Pandan	18.21 ± 0.35 h	0.80 ± 0.14 l	19.11 ± 1.02 d
Stevia	65.50 ± 5.74 c	22.92 ± 0.57 b	26.88 ± 1.80 b
Lemongrass	10.77 ± 0.93 ijk	1.69 ± 0.41 l	10.78 ± 2.02 fg
Asiatic pennywort	12.58 ± 0.40 ij	10.73 ± 0.46 efg	11.89 ± 2.24 f
Jiaogulan	5.02 ± 1.32 l	1.19 ± 0.50 l	2.09 ± 0.73 l
Kariyat	18.51 ± 1.62 gh	7.66 ± 0.96 ghi	8.39 ± 0.69 hi
Mulberry leave	19.99 ± 1.64 fgh	6.24 ± 1.39 ijk	8.02 ± 0.32 hi
Cat’s whiskers	24.78 ± 2.78 ef	3.45 ± 0.23 k	9.86 ± 1.04 g
Bamboo grass	10.12 ± 0.22 jk	8.84 ± 0.61 fghi	32.50 ± 1.15 a
Sea holly	10.51 ± 1.48 ijk	13.48 ± 0.54 de	14.47 ± 0.58 e
Stonebreaker	102.39 ± 2.67 a	19.93 ± 0.45 bc	18.18 ± 0.36 d
Jewel vine	37.09 ± 2.33 d	14.21 ± 2.88 d	1.61 ± 0.64 l
Safflower	24.78 ± 2.78 ef	3.45 ± 0.23 k	9.86 ± 1.04 g
Chrysanthemum	29.07 ± 1.87 e	12.08 ± 1.71 def	27.79 ± 2.10 b
Roselle	6.24 ± 1.20 kl	3.37 ± 0.53 kl	5.25 ± 1.30 k
Butterfly pea	23.95 ± 0.51 efg	3.95 ± 0.66 jk	9.17 ± 1.00 ghi
Beal fruit	101.38 ± 6.10 a	17.98 ± 1.89 c	17.34 ± 0.49 d
Indian gooseberry	81.86 ± 7.39 b	6.78 ± 0.00 hij	23.85 ± 0.92 c
Bitter gourd	11.65 ± 1.08 ijk	2.30 ± 0.50 l	14.57 ± 0.68 e
Siamese senna	64.74 ± 3.75 c	43.50 ± 2.36 a	12.10 ± 0.93 f
Chinese liquorice	25.52 ± 0.23 ef	8.29 ± 0.62 gh	7.36 ± 0.49 ij
Alexandria senna	27.49 ± 0.57 e	10.08 ± 1.43 fgh	5.55 ± 1.49 jk
Ginger	9.35 ± 1.76 jk	3.41 ± 0.56 kl	15.24 ± 1.25 e
Ginkgo	16.13 ± 0.17 hi	2.32 ± 0.77 l	2.89 ± 0.80 l
Black galingale	8.93 ± 0.22 jk	1.67 ± 0.58 l	14.35 ± 0.35 e

Values are means ± SD of three measurements (n = 3).

Means in columns with superscript letters indicate significant differences (*p* < 0.05).

### Total flavonoid content

The contents of total flavonoid in herbal infusions were significantly different (*p* < 0.05) and comparable (Table 3). The content of flavonoids in herbal infusions, ranged from 0.80 mg QE/g DW (pandan) to 43.50 mg QE/g DW (Siamese senna) with a difference of 54-fold, and the mean value was 9.6 mg QE/g DW for 25 herbs studied. The higher total flavonoid content was observed in stonebreaker (19.93 mg QE/g DW) and beal fruit (17.98 mg QE/g DW), respectively. The flavonoid content of in Indian gooseberry extracts varied between 0.71 mg QE/g DW and 10.34 mg QE/g DW^18^. Total flavonoids of 13.6 g/100 g DW in green and 4.2 g/100 g DW in black tea was reported^21^. It indicated that the herbal infusions in our study might contribute the same health benefit as those in tea in terms of polyphenols.

### DPPH radical scavenging activity

Table 3 shows a significant difference (*p* < 0.05) in the results of antioxidant activity determined by using DPPH radical scavenging assay. The DPPH inhibition varied from 1.61% to 32.50% with a difference of 20-fold, and the mean value was 14.24% for the 25 herbal tea infusions. Bamboo grass inhibited 32.50% of the oxidation while jewel vine showed the lowest DPPH inhibition (1.61%). Moreover, the DPPH inhibition was observed in chrysanthemum (27.79%), followed by stevia (26.88%), Indian gooseberry (23.85%), pandan (19.11%) and stonebreaker (18.18%), respectively. The DPPH inhibition of Indian gooseberry extracted with difference solvents ranged from 13.64% to 83.14% was reported^18^.

### α-amylase inhibition

**Figure 2:**
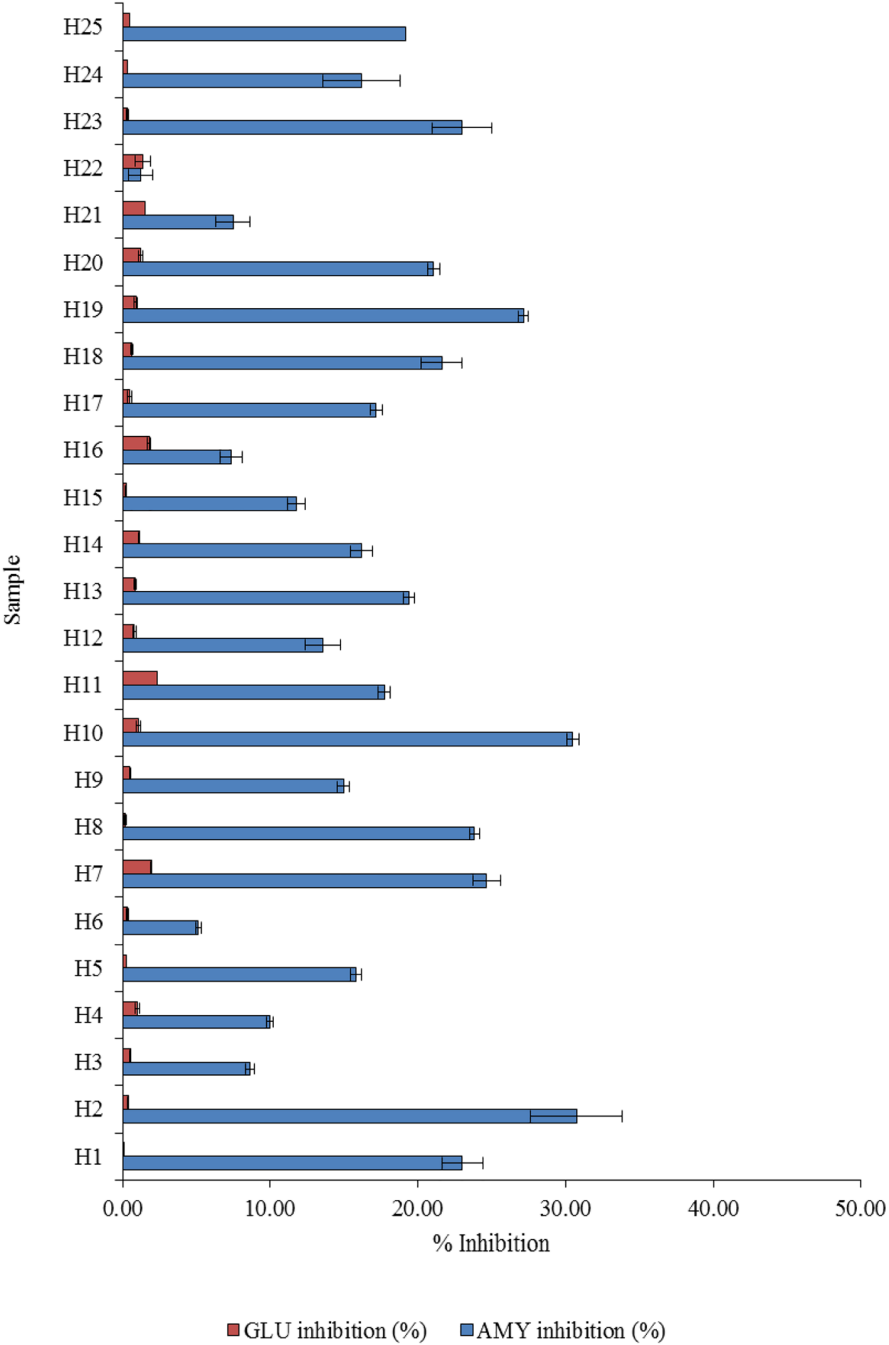
In vitro α-amylase (AMY) and α-glucosidase (GLU) inhibition of herbal infusions.

A significant difference in the potential inhibition against α-amylase (*p* < 0.05) was observed (Fig. 2). For the herbal infusions studied, the inhibitory activities toward α-amylase were in a wide range between 1.23% (*Alexandria senna*) and 30.75% (stevia) and the average value was 17.09% for the 25 herbal infusions. However, in our study, the beal fruit and stonebreaker infusions containing the highest total phenolic content (Table 3) showed the α-amylase inhibition of 17.17% and 17.73%, respectively. In addition, dried pandan, Indian gooseberry and stonebreaker extracts containing a similar main dominant-vibrational spectrum of chemical compositions (Fig. 1.), had the α-amylase inhibition with 22.99%, 21.61% and 17.73%, respectively.

### α-glucosidase inhibition

Although the herbal plants appeared to have anti-α-glucosidase activities^7,22^ our experiments showed that the studied herbal infusions are a weak inhibition against α-glucosidase. Figure 2 shows the α-glucosidase inhibition varied from 0.09% (pandan) to 2.38% (stonebreaker). The infusion of stonebreaker showed the highest α-glucosidase inhibition.

### Lipase inhibition

**Figure 3:**
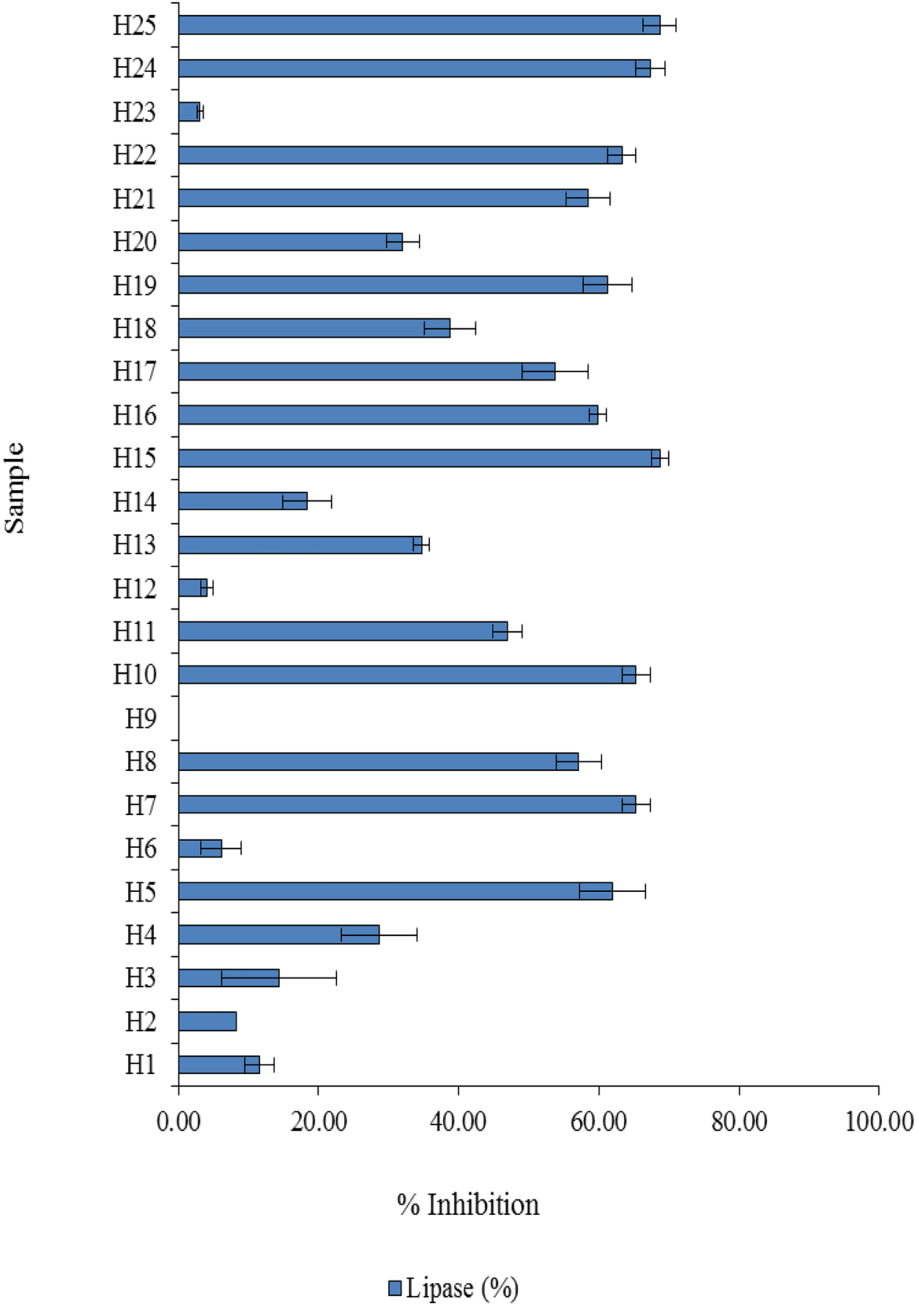
In vitro lipase inhibition of herbal tea infusions.

Among the herbal infusions examined, only the bamboo grass infusion did not show its inhibitory activity against lipase. A wide range of lipase inhibition, from 0.00% to 68.71% was observed (Fig. 3). A relatively high inhibitory activity against lipase with a percentage higher than 65% was observed in many herbal infusions—for example, roselle, ginkgo, mulberry, sea holly and black galingale.

### Pearson’s correlation coefficient

Table S3 in the Supplement information shows the Pearson’s correlation coefficient among phenolic compositions, total phenolic, total flavonoid contents and the inhibition of α-amylase and α-glucosidase and lipase, respectively. A high correlation (*R*^2^ = 0.63, *p* < 0.05) was observed between total phenolic and total flavonoid contents, while a high correlation (*R*^2^ = 0.58, *p* < 0.05) was also observed between total phenolic and gallic acid contents. The correlations between α-amylase inhibition and phenolic compositions were from − 0.37 to 0.13; between α-glucosidase inhibition and phenolic compositions from 0.21 to 0.54; and between lipase inhibition and phenolic compositions they were from − 0.33 to 0.16. However, a minimal correlation (*R*^2^ = 0.19 and *R*^2^ = 0.26, *p* < 0.05) was observed between total phenolic and total flavonoid contents and α-amylase inhibition, respectively, while a minimal correlation (*R*^2^ = 0.28 and *R*^2^ = 0.23, *p* < 0.05) was also observed between total phenolic and total flavonoid contents and α-glucosidase inhibition, respectively. Therefore, the contribution of phenolic compositions in herbal infusions to α-amylase and α-glucosidase inhibition might be resulted from synergy effects and not caused by an individual phenolic compound^23^. However, there was no significant relation between total phenolic, total flavonoid contents, phenolic compositions and lipase inhibition (*p* ≥ 0.05).

## Conclusions

The qualitative analysis of dried herbal plant extracts using FT-IR spectroscopy provided fast measurements. The vibrational spectra of dried plant extracts were highly related to the presence of aromatic compounds in the plants. In addition, a quantitative analysis of major individual phenolics in the herbal infusions was performed to explain the relationships between potential health benefits and phenolic compositions. Pandan, Indian gooseberry and stonebreaker show the similar FT-IR vibrational spectra and might be a good source of phenolic compounds and a potent natural phenolic antioxidant for early hyperglycemic management. A positive and significant correlation existed between *p*-coumaric acid and α-glucosidase inhibition and antioxidant activity and α-amylase inhibition, measured by in vitro analysis, revealing the contribution of phenolic compounds to potential health benefits of plants. An in vivo study of these herbal tea infusions on diabetes and obesity should be further performed.

## Materials and methods

Twenty-five kinds of dried herbal teas, pre-packed in a tea bag, were purchased from local supermarkets in Chiang Rai province, Thailand in 2019. Shelf-life of herbal teas was 2 months old. The cultivars were accepting the terms and conditions of the Standard Material Transfer Agreement (SMTA) of the International Treaty on Plant Genetic Resources for Food and Agriculture. The use of plants in the present study complied with the Mae Fah Luang University ethic, National Research Council of Thailand ethic and international institutional guidelines. The samples were coded using ordinary number codes, as shown in the Supplement Information Table S1. The shelf-life of products was less than 3 months. The samples were stored at 25 °C until further analysis.

### Chemicals

Folin-Ciocalteu phenol reagent (FC), 2, 2-diphenyl-1-picrylhydrazyl (DPPH), porcine pancreatic α-amylase (EC 3.2.1.1), *Saccharomyces cerevisiae* α-glucosidase (EC 3.2.1.20), and porcine pancreas lipase (EC 3.1.1.3) were purchased from Sigma-Aldrich (St. Louis, MO, USA). All other chemicals were of analytical reagent grade. Authentic phenolic standards including caffeic, *p-*coumaric, ferulic and gallic acids, quercetin and (+)-catechin were obtained from Sigma-Aldrich (St. Louis, MO, USA). HPLC-grade methanol, acetonitrile and other solvents and reagents were purchased from Merck & Co. Inc. (NJ. USA) Deionized water prepared by Millipore purification system (Millipore Corp. Marlborough, MA, USA) was used for preparation of solutions.

### Preparation of plant samples

#### Preparation of herbal tea infusions per serving

Three grams (one tea bag) of each sample were infused into 150 mL of boiling deionized water (equivalent to a tea cup) for 5 min prior to being filtered through Whatman No.2 filter paper. The filtrate then was used to analyse for total phenolic and flavonoid contents, antioxidant activity, α-amylase, α-glucosidase and lipase inhibition activities.

#### Freeze drying

Five grams of each sample were extracted into 100 mL of boiled deionized water for 5 min before being filtered through a Whatman No.2 filter paper and centrifuged at 4000 g (VS15000N, Vision Scientific Co. Ltd. Korea) for 15 min. The supernatant was dried by using a freeze dryer (Martin Christ Gefriertrocknungsanlagen GmbH, Osterode am Harz, Germany). The freeze-dried samples were used to characterize metabolite compounds by using FT-IR spectroscopy and determine phenolic compounds by using UPLC analysis.

#### FT-IR measurements and spectral collection

Ten milligrams of each freeze-dried extract were dispersed and encapsulated in 100 mg of potassium bromide (KBr) to form a thin translucent sample disc for FT-IR analysis. The disc was then placed in a sample cup of a diffuse reflectance accessory. FT-IR experiments were performed using PerkinElmer 2000 infrared spectrometer (PerkinElmer, Inc. MA, USA) to identify the characteristic functional groups in the sample. The spectra were measured by Spectrum 10.03.09.0139 software® (PerkinElmer Inc., MA, USA) and recorded within a range of 4000–400 cm^−1^, with a resolution of 4 cm^−1^.

### Determination of phenolic profile

The targeted phenolic compound was determined by using the method of Spáčil^11^ with some modifications. Five hundred milligrams of each freeze-dried sample were dissolved by mixing with 5 mL of warm distilled water (80 °C), gently shaken for 5 min and filtered through Whatman No.2 filter paper prior to filtrate through a 0.2 µm Millipore filter. A total of 1.0 µL of the filtered extract was injected to a UPLC Acquity™ system (Waters Pacific Pte Ltd, Singapore) coupled to a tunable UV detector and an autosampler. All UPLC analyses were conducted on a bridged ethylene hybrid (BEH) C_18_ analytical column (100 mm × 2.1 mm, 1.7 µm, Waters Pacific Pte Ltd, Singapore) and temperature of separation was set constant at to 25 °C. The 0.05% trifluoroacetic acid (TFA) in methanol (mobile phase A) and 100% methanol (mobile phase B) was applied for the gradient elution. The ratio of mobile phases was dependent on the gradient profile at a flow rate of 1 mL per min at room temperature.

Chromatographic data were acquired and integrated using Waters’ Empower™ 2 software (Waters Pacific Pte Ltd., Singapore). Spectra were recorded from 200 to 600 nm. The qualification and quantification of caffeic, *p*-coumaric, ferulic and gallic acids were carried out at 280 nm while of flavonols, including (+)-catechin and quercetin were conducted at 370 nm.

The retention times and spectral characteristics of phenolic compounds in the samples were identified and compared with those of reference standards. The calibration curve of standard compounds including caffeic, *p*-coumaric, ferulic and gallic acids, quercetin and (+)-catechin were prepared by dissolving 5 mg of standard compounds in 10 mL of HPLC-grade methanol. The standard concentration ranged from 10 to 125 µg/mL. The content of phenolic compounds was quantified and expressed as mg/g dry weight (DW).

### Determination of total phenolic content (TPC)

The total phenolic content (TPC) was determined according to the International Organization for Standardization (ISO) (2005)^12^ with some modifications. A 1 mL of each infusion was incubated with 5 mL of 10% (v/v) freshly prepared Folin-Ciocalteu reagent in distilled water for 5 min at room temperature. A 4 mL of 7.5% (w/v) sodium carbonate (Na_2_CO_3_) solution then was added and mixed well prior to the reaction mixture was re-incubated in the dark at room temperature for 60 min. The absorbance of the resulting mixture was measured at 765 nm using a UV/Vis spectrophotometer (Biochrom-Libra S22, Cambridge, UK). A calibration standard was prepared by using gallic acid solution range between 10 and 50 µg/mL. The content of phenols in the samples were calculated and expressed as mg gallic acid equivalents in 1 g of dried sample (mg GAE/g DW).

### Determination of total flavonoid content (TFC)

The TFC was determined by using the aluminium chloride method described by Abu Bakar, Mohamed, Rahmat and Fry (2009)^13^ with slight modifications. A 0.5 mL of each infusion was dissolved in 2.25 mL of distilled water prior to 0.15 mL of 5% NaNO_2_ solution being added and mixed well. After 6 min incubation, 0.3 mL of a 10% AlCl_3_.6H_2_O solution was added and allowed to stand for another 5 min before 1.0 mL of 1 M NaOH was added and mixed well. The absorbance of the resulting mixture was measured immediately at 510 nm using a UV/Vis spectrophotometer (Biochrom-Libra S22, Cambridge, UK). A varied concentration of quercetin was prepared and used for a calibration curve. The concentration of flavonoid was then calculated and expressed as mg quercetin equivalents in 1 g of dried sample (mg QE/g DW).

### Determination of DPPH radical scavenging activity

The DPPH radical scavenging activity was determined by using Ranilla et al.’s method^7^ with slight modifications. Briefly, a 50 µL of each infusion was incubated with 1.95 mL of DPPH in solution (6 × 10^–5^ M in methanol). After 30 min incubation in the dark at room temperature, the absorbance at 517 nm was recorded using a UV/Vis spectrophotometer (Biochrom-Libra S22, Cambridge, UK). The inhibitory activity was expressed as a percentage of a control sample without the infusions using the following equation:

1 $$\% {\text{ inhibition }} = {\text{ A517 }}\left( {{\text{control}}} \right) \, {-}{\text{ A517 }}({\text{sample}})/{\text{A517 }}\left( {{\text{control}}} \right) \times {1}00$$

where A517 (control) = absorbance without sample; A517 (sample) = absorbance with sample.

### Determination of α-amylase inhibitory activity

The α-amylase inhibition activity was determined according to the method described by Ranilla et al.’s method^7^ with some modifications. Herbal infusions (500 μL) were mixed 500 μL of 0.02 M sodium phosphate buffer, pH 6.9 with 0.006 M sodium chloride containing 0.5 mg/mL α-amylase solution. The reaction mixtures were incubated at 25 °C in a water bath for 10 min. A 1% (w/v) starch solution in 0.02 M sodium phosphate buffer (pH 6.9 with 0.006 M NaCl) was prepared. A 500 µL of 1% (w/v) starch solution was added to the reaction mixture and re-incubated at 25 °C in a water bath for 10 min. A 1.0 mL of dinitrosalicyclic acid (DNSA) colour forming reagent was then added and boiled in a boiling water bath for 7 min to terminate the reaction. Finally, 1.0 mL of 18.2% tartrate solution was added and cooled at room temperature. The reaction mixture was diluted with 10 ml of distilled water. The absorbance of the resulting mixture was measured at 540 nm with a UV/Vis spectrophotometer (Biochrom-Libra S22, UK). The α-amylase inhibition was calculated and expressed as a percentage of control sample without the infusions by using the following equation:

2 $$\% {\text{ inhibition }} = {\text{ A54}}0 \, \left( {{\text{control}}} \right) \, - {\text{ A54}}0 \, ({\text{sample}})/{\text{A54}}0 \, \left( {{\text{control}}} \right) \times {1}00$$

where: A540 (control) = absorbance without sample; A540 (extract) = absorbance with sample.

### Determination of α-glucosidase inhibitory activity

The inhibition of α-glucosidase was determined by using Ranilla et al.’s method^7^ method (2010), with some modifications. α-Glucosidase (1000 U) was dissolved in 100 mL of 0.1 M potassium phosphate buffer (pH 6.9). Aliquots of 500 μL were made and these were stored at – 20 °C. Each ampoule contained 5 U/500 μL of α-glucosidase enzyme solution. Consequently, an aliquot of 500 µL of each infusion was incubated with 1 mL of α-glucosidase solution (1.0 U/mL) in 0.1 M potassium phosphate buffer (pH 6.9) at 25 °C in a warm water bath for 10 min. Potassium phosphate buffer (0.1 M, pH 6.9) was added into 5 mM *p*-nitrophenyl-α-D-glucopyranoside under continuous stirring to ensure thorough mixing. Thereafter, a 500 µL of *p*-nitro phenyl-α-D-glucopyranoside solution (5 mM) in 0.1 M potassium phosphate buffer (pH 6.9) was added, and the reaction mixture was further incubated at 25 °C in a warm water bath for 5 min. The absorbance of the released *p*-nitrophenol was measured at 405 nm using a UV/Vis spectrophotometer (Biochrom-Libra S22, UK), before and after the incubation period. The percentage of α-glucosidase inhibition was calculated by using the following equation:

3 $$\% {\text{ inhibition }} = (\Delta {\text{A4}}0{5 }\left( {{\text{control}}} \right) \, - \, \Delta {\text{A4}}0{5 }({\text{sample}})/\Delta {\text{A4}}0{5 }\left( {{\text{control}}} \right){\text{x1}}00$$

where: ΔA405 (control) = absorbance without sample; ΔA405 (sample) = absorbance with sample.

### Determination of lipase inhibitory activity

The inhibition of lipase was determined by use of Gondoin et al.’s method with some modifications^9^. An aliquot of 10 mg/mL of lipase was prepared by dissolving porcine pancreas in ultra-pure water. The mixture was then centrifuged at 16,000*g* for 5 min by using a microcentrifuge (VS15000N, Vision Scientific Co. Ltd. Korea). The substrate of reaction was prepared by dissolving 0.08% (w/v) 4-nitrophenyl laurate (*p*-NP laurate) in 1% (w/v) Triton X-100 in 5 mM sodium acetate (pH 5.0). The reaction mixture was then boiled in a boiling water bath for 1 min to aid dissolution, mixed well, and then cooled to room temperature. In addition, a 100 mM Tris buffer (pH 8.2) was used as the assay buffer. An aliquot of 50 µL each infusion was mixed with 150 µL lipase and 400 µL assay buffer. Subsequently, a 450 µL of substrate was added to start the reaction. Following 2-h incubation at 37 °C, the reaction mixture was centrifuged at 16,000*g* for 5 min by using a microcentrifuge (VS15000N, Vision Scientific Co. Ltd. Korea). The absorbance of supernatant was measured at 400 nm by using a UV/Vis spectrophotometer (Biochrom-Libra S22, Cambridge, UK). The percentage of lipase inhibition was calculated by using the following equation:

4 $$\% {\text{ inhibition }} = {\text{ A4}}00 \, \left( {{\text{control}}} \right) \, - {\text{ A4}}00 \, ({\text{sample}})/{\text{A4}}00 \, \left( {{\text{control}}} \right) \times {1}00$$

where: A400 (control) = absorbance without sample; A400 (extract) = absorbance with sample.

### Statistical analysis

All experiments were carried out in triplicate. Results were presented as means ± standard deviation (n = 3). Data were statistically analysed by SPSS software package version 15.0.1 (SPSS: An IBM Company, NY, USA) using one-way analysis of variance (ANOVA) and Duncan’s multiple-range test procedures. Values were considered significantly difference at *p* < 0.05. Pearson Correlation test is used to evaluate the association between phenolic profiles and inhibitory potential against digestive enzymes. The Pearson’s correlation, *R*^2^, demonstrated how the response variables were related mathematically and to understand the proportion of the variance of one variable that is predictable from the other variable.

## Supplementary Information

Supplementary Tables.
