# Supplementary material for: FT-IR characteristics, phenolic profiles and inhibitory potential against digestive enzymes of 25 herbal infusions
Source: Sci Rep. 2022 Apr 22;12:6631. doi: 10.1038/s41598-022-10669-z (PMC9033800; doi:10.1038/s41598-022-10669-z)
Supplement: Supplementary file 1 — Supplementary Tables. [file 41598_2022_10669_MOESM1_ESM.doc]

**Supplement Information**

Table S1 Herbal infusions

| No. | Sample code | Herb | Scientific name | Family | Edible part |
| --- | --- | --- | --- | --- | --- |
| 1. | H1 | Pandan | *Pandanus amaryllifolius* Roxb. | *Pandanaceae* | Leaves |
| 2. | H2 | Stevia | *Stevia rebaudiana* Bertoni | *Asteraceae* | Leaves |
| 3. | H3 | Lemongrass | *Cymbopogon citratus* | *Poaceae* | Leaves |
| 4. | H4 | Asiatic pennywort | *Centella asiatica* | Apiaceae | Leaves |
| 5. | H5 | Jiaogulan | *Gynostemma pentaphyllum* (Thunb.) Makino | Cucurbitaceae | Leaves |
| 6. | H6 | Kariyat | *Andrographis paniculata* (Burm.f.) Nees | *Acanthaceae* | Leaves |
| 7. | H7 | Mulberry leave | *Morus alba* Linn. | *Moraceae* | Leaves |
| 8. | H8 | Cat’s whiskers | *Orthosiphon aristatus* | *Lamiaceae* | Leaves |
| 9. | H9 | Bamboo grass | *Tiliacora triandra* | *Menispermaceae* | Leaves |

Table S1 (Continued) Herbal infusions

| No. | Sample code | Herb | Scientific name | Family | Edible part |
| --- | --- | --- | --- | --- | --- |
| 10. | H10 | Sea holly | *Acanthus ebracteatus* | *Acanthaceae* |  |
| 11. | H11 | Stonebreaker | *Phyllanthus niruri* L. | *Phyllanthaceae* |  |
| 12. | H12 | Jewel vine | *Derris scandens* (Roxb.) Benth. | *Fabaceae* |  |
| 13. | H13 | Safflower | *Carthamus tinctorius* | *Asteraceae* |  |
| 14. | H14 | Chrysanthemum | *Chrysanthemum indicum* Linn. | *Compositae* |  |
| 15. | H15 | Roselle | *Hibiscus sabdariffa* Linn. | *Malvaceae* |  |
| 16. | H16 | Butterfly pea | *Clitoria ternatea* L. | *Fabaceae* |  |
| 17. | H17 | Beal fruit | Aegle marmelos (L.) Corrêa | *Rutaceae* | Fruit |

Table S1 (Continued) Herbal infusions

| No. | Sample code | Herb | Scientific name | Family | Edible part |
| --- | --- | --- | --- | --- | --- |
| 18. | H18 | Indian gooseberry | *Phyllanthus emblica* | *Phyllanthaceae* | Fruit |
| 19. | H19 | Bitter gourd | *Momordica charantia* L. | *Cucurbitaceae* | Fruit |
| 20. | H20 | Siamese senna | *Senna siamea* (Lam.) H.S.Irwin & Barneby | *Fabaceae* | Fruit |
| 21. | H21 | Chinese liquorice | *Glycyrrhiza glabra* L. | *Fabaceae* | Fruit |
| 22. | H22 | Alexandria senna | *Senna alexandrina* Mill. | *Fabaceae* | Fruit |
| 23. | H23 | Ginger | Zingiber officinale | *Zingiberaceae* | Rhizome |
| 24. | H24 | Ginkgo | *Ginkgo biloba* | *Ginkgoaceae* | Rhizome |
| 25. | H25 | Black galingale | *Kaempferia parviflora* Wallich. ex Baker. | *Zingiberaceae* | Rhizome |

Table S2 Absorption spectra of function groups

| Origin | Group frequency (wavenumber, cm^-1^) | Functional group assignment | References |
| --- | --- | --- | --- |
| Saturated aliphatic (alkane/alkyl) group frequencies | | | |
| Methyl (−CH_3_) | | | |
|  | 2970–2950/2880–2860 | Methyl C-H asym. /sym. stretch | [14,16] |
|  | 1470–1430/1380–1370 | Methyl C-H asym. /sym. bend |
| Methylene (-CH2) | | | |
|  | 2935–2915/2865–2845 | Methylene C-H asym. /sym. stretch | [14,16] |
|  | 1485–1445 | Methylene C-H bend |
| Methyne (−CH−) | | | |
|  | 2900–2880 | Methyne C-H stretch | [14,16] |
|  | 1350–1330 | Methyne C-H bend |
|  | 1300–700 | Skeletal C-C vibrations |
| Olefinic (alkene) group frequencies | | | |
| C=C | 1680–1620 | Alkenyl C=C stretch | [14,16] |
|  | 1625 | Aryl-substituted C=C |
|  | 1600 | Conjugated C=C |
| C-H | 3095–3075 + 3040–3010 | Terminal (vinyl) C-H stretch |
| C-H | 970–960 | *trans*- C-H out-of-plane bend |
|  | 700 (broad) | *cis*- C-H out-of-plane bend |
| Aromatic ring (aryl) group frequencies | | | |
| C=C-C^a^ | 1615–1580 | Aromatic ring stretch | [14,15,16] |
| C=C-C^a^ | 1510–1450 | Aromatic ring stretch |
| C-H | 1225–950 (several) | Aromatic C-H in-plane bend |
| C-H | 900–670 (several) | Aromatic C-H out-of-plane bend |
| ‘Combi’’^b^ | 2000–1660 (several) | Aromatic combination bands |
| Hydroxy compound group frequencies | | | |
| O-H | 3570–3200 (broad) | Hydroxy group, H-bonded; O-H stretch | [14,15,16] |
|  | 3400–3200 | Normal ‘‘polymeric’ O-H stretch |
|  | 3550–3450 | Dimeric O-H stretch |
|  | 3570–3540 | Internally bonded O-H stretch |
| O-H | 3645–3600 (narrow) | Non-bonded hydroxy group, O-H stretch |
|  | 3640–3530^c^ | Phenols, O-H stretch |
| O-H | 1410–1310 | Phenols, O-H bend |
| C-O | ~1200^d^ | Phenol, C-O stretch |
|  |  |  |

^a^ C=C-C^a^ used as an approximation of the unique aromatic ring bonding.

^b^ ‘‘Combi’’ denotes assignment to combination bands.

^c^ Frequency influenced by nature and position of other ring substituents.

^d^ Approximate center of range for the group frequency.

|  |
| --- |

Table S3 Pearson’s correlation

|  | TPC | TFC | GAL | CAT | CAF | COU | FER | QUE | DPPH | AMY | GLU | LIP |
| --- | --- | --- | --- | --- | --- | --- | --- | --- | --- | --- | --- | --- |
| TPC | 1 | 0.63 | 0.58 | 0.40 | 0.08 | 0.35 | 0.17 | -0.16 | 0.31 | 0.19 | 0.28 | -0.05 |
| TFC |  | 1 | 0.12 | 0.18 | 0.13 | 0.00 | 0.51 | -0.13 | 0.28 | 0.26 | 0.23 | -0.16 |
| GAL |  |  | 1 | 0.77 | 0.06 | 0.63 | 0.00 | -0.04 | 0.07 | -0.01 | 0.48 | 0.06 |
| CAT |  |  |  | 1 | -0.04 | 0.34 | -0.10 | 0.47 | 0.10 | 0.13 | 0.37 | -0.18 |
| CAF |  |  |  |  | 1 | 0.16 | 0.77 | -0.07 | -0.20 | -0.37 | 0.28 | 0.16 |
| COU |  |  |  |  |  | 1 | 0.09 | -0.12 | -0.13 | -0.34 | 0.54 | 0.12 |
| FER |  |  |  |  |  |  | 1 | -0.09 | -0.08 | -0.16 | 0.21 | 0.01 |
| QUE |  |  |  |  |  |  |  | 1 | 0.03 | 0.16 | -0.18 | -0.33 |
| DPPH |  |  |  |  |  |  |  |  | 1 | 0.46 | -0.17 | -0.32 |
| AMY |  |  |  |  |  |  |  |  |  | 1 | -0.16 | 0.01 |
| GLU |  |  |  |  |  |  |  |  |  |  | 1 | 0.28 |
| LIP |  |  |  |  |  |  |  |  |  |  |  | 1 |

Notes: TPC: total phenolic content; TFC: total flavonoid content; Gal: gallic acid; CAF: caffeic acid; CAT: catechin; COU: *p*-coumaric acid; FER: ferulic acid; QUE: quercetin; DPPH: DPPH inhibition; AMY: α-amylase inhibition; GLU: α-glucosidase inhibition; LIP: lipase inhibition.
